# Catalytic Hydrogenation of the Sweet Principles of *Stevia rebaudiana*, Rebaudioside B, Rebaudioside C, and Rebaudioside D and Sensory Evaluation of Their Reduced Derivatives

**DOI:** 10.3390/ijms131115126

**Published:** 2012-11-16

**Authors:** Indra Prakash, Mary Campbell, Venkata Sai Prakash Chaturvedula

**Affiliations:** 1 Organic Chemistry Department, Global Research and Development, The Coca-Cola Company, One Coca-Cola Plaza, Atlanta, GA 30313, USA; E-Mail: iprakash@coca-cola.com; 2 Product Development, Coca-Cola North America Division, The Coca-Cola Company, One Coca-Cola Plaza, Atlanta, GA 30313, USA; E-Mail: marycampbell@coca-cola.com

**Keywords:** *ent*-kaurane diterpene glycosides, catalytic hydrogenation, Pd(OH)_2_, structure characterization, spectral data, sensory evaluation

## Abstract

Catalytic hydrogenation of rebaudioside B, rebaudioside C, and rebaudioside D; the three *ent*-kaurane diterpene glycosides isolated from *Stevia rebaudiana* was carried out using Pd(OH)_2_. Reduction of steviol glycosides was performed using straightforward synthetic chemistry with the catalyst Pd(OH)_2_ and structures of the corresponding dihydro derivatives were characterized on the basis of 1D and 2D nuclear magnetic resonance (NMR) spectral data indicating that all are novel compounds being reported for the first time. Also, the taste properties of all reduced compounds were evaluated against their corresponding original steviol glycosides and sucrose.

## 1. Introduction

**Figure 1:**
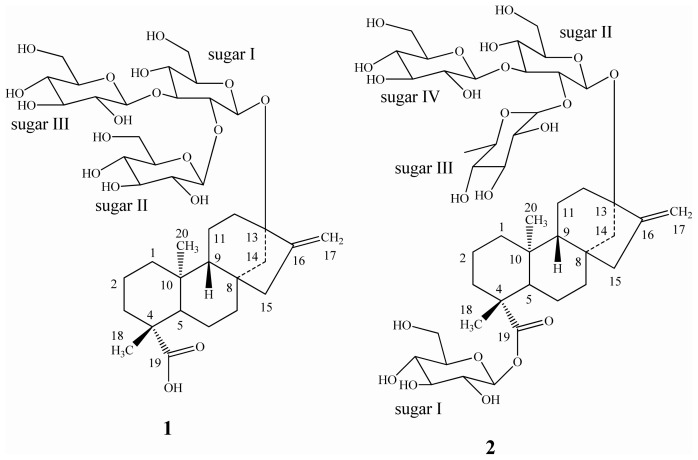

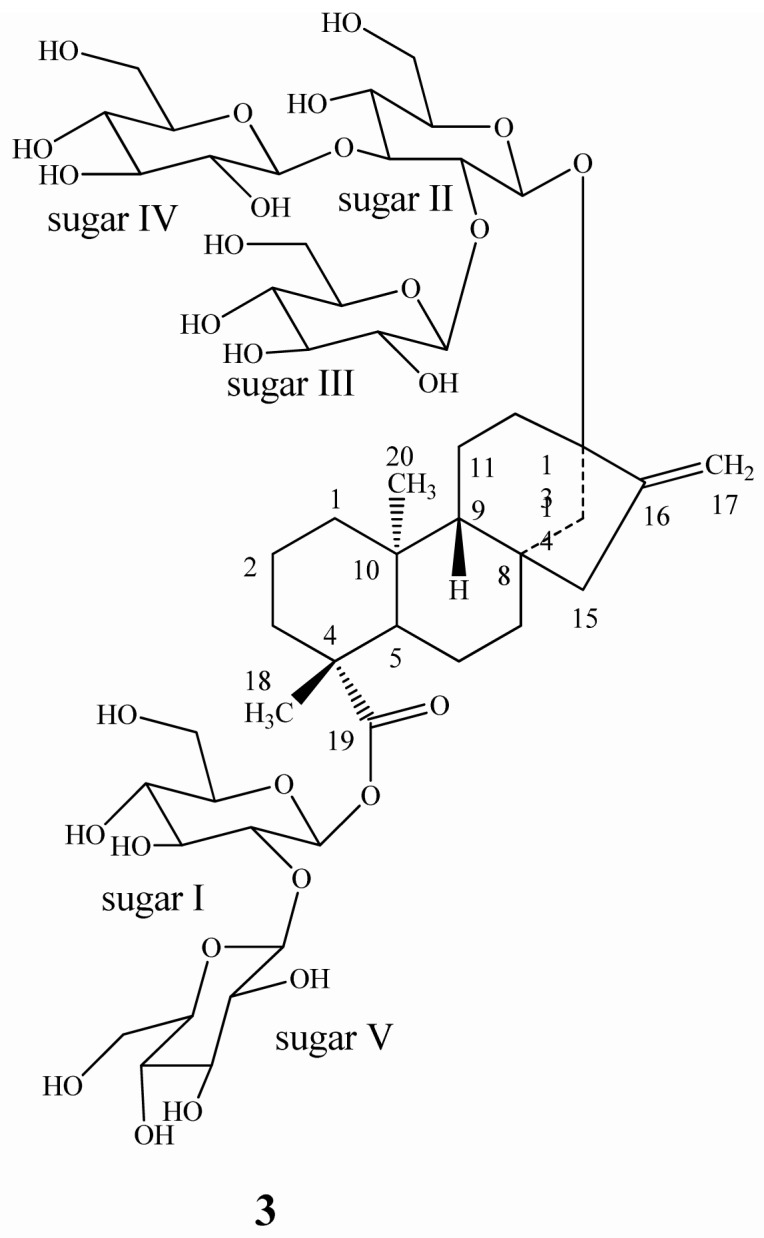
Structures of rebaudioside B (**1**), rebaudioside C (**2**), and rebaudioside D (**3**).

Rebaudioside B, rebaudioside C, and rebaudioside D are the minor constituents isolated from the leaves of *Stevia rebaudiana* Bertoni (family: Asteraceae). These compounds are the glycosides of the diterpene steviol, *ent*-13-hydroxykaur-16-en-19-oic acid [1]. Rebaudioside B tastes about 150 times sweeter than sucrose; rebaudioside C tastes about 20–30 times sweeter than sucrose, and rebaudioside D tastes about 200–220 times sweeter than sucrose; all are non-caloric. Rebaudioside B (**1**) has a (2-*O*-β-d-glucopyranosyl-3-*O*-β-d-glucopyranosyl)-β-d-glucopyranosyl unit at the *C*-13 position with a free carboxylic acid group at the *C*-19 position of the aglycone steviol; whereas rebaudioside C (**2**) has a (2-*O*-α-l-rhamnopyranosyl-3-*O*-β-d-glucopyranosyl)-β-d-glucopyranosyl unit at the *C*-13 position and a β-d-glucosyl moiety at the *C*-19 position of the aglycone steviol in the form of an ester; and rebaudioside D (**3**) has a (2-*O*-β-d-glucopyranosyl-3-*O*-β-d-glucopyranosyl)-β-d-glucopyranosyl unit at the *C*-13 position and a 2-*O*-β-d-glucopyranosyl-β-d-glucopyranosyl moiety at the *C*-19 position of the aglycone steviol as an ester (Figure 1). As a part of our continuing research to discover natural sweeteners, we have reported several glycosides from the commercial extract of *S. rebaudiana*[2–9]. Apart from isolating novel compounds from *S. rebaudiana* and utilizing them as possible natural sweeteners or sweetness enhancers, we are also engaged in understanding the physicochemical profiles of steviol glycosides in various systems of interest and structural characterization of their metabolites as well as their synthesis [10–13]. Recently, we have published the catalytic reduction of the three *ent*-kaurane diterpene glycosides namely rubusoside, stevioside, and rebaudioside A isolated from *S. rebaudiana* and their sensory evaluation [14]. In this article, we present the synthesis of novel *ent*-kaurane diterpene glycosides that are prepared by reduction of their *C*-16/*C*-17 exocyclic double bond of rebaudioside B, rebaudioside C, and rebaudioside D; their structures were characterized on the basis of extensive nuclear magnetic resonance (NMR) and mass spectroscopic (MS) data as well as enzymatic hydrolysis studies.

## 2. Results and Discussion

### 2.1. Catalytic Hydrogenation of Rebaudioside B (**1**), Rebaudioside C (**2**), and Rebaudioside D (**3**)

**Scheme 1:**
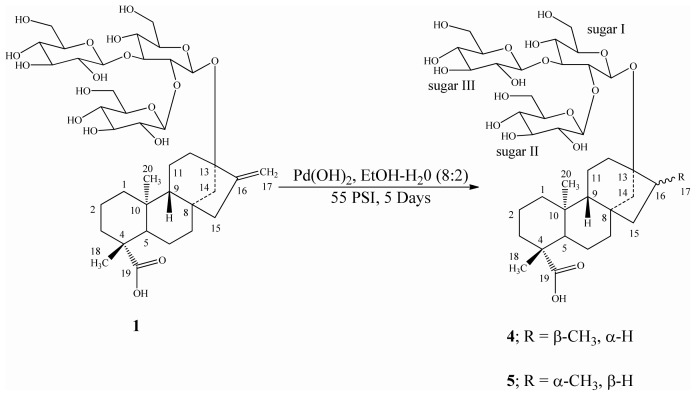
Hydrogenation of rebaudioside B (**1**) and its reduced compounds.

**Scheme 2:**
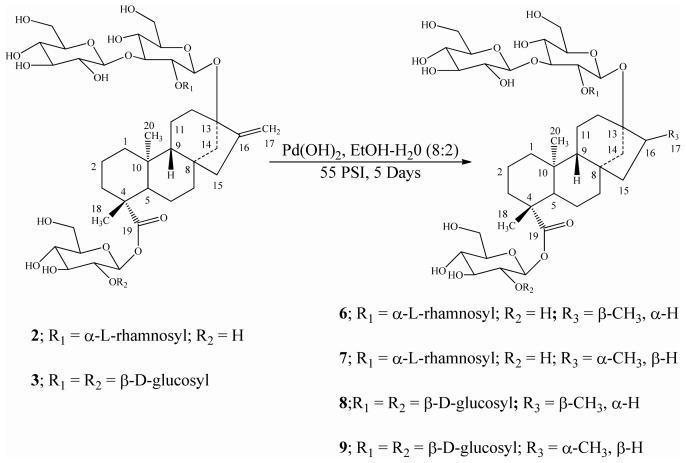
Hydrogenation of rebaudioside C (**2**) and rebaudioside D (**3**) and their reduced compounds.

Reduction of the three *ent*-kaurane diterpene glycosides isolated from *S. rebaudiana* namely rebaudioside B (**1**), rebaudioside C (**2**), and rebaudioside D (**3**) was performed using catalytic hydrogenation with Pd(OH)_2_ in a solvent mixture of EtOH/H_2_O (8:2) at room temperature under 55 psi H_2_ as reported earlier [14], which furnished mixtures of dihydrorebaudioside-B 1/2 (**4**/**5**) (Scheme 1), dihydrorebaudioside-C 1/2 (**6**/**7**), and dihydrorebaudioside-D 1/2 (**8**/**9**) (Scheme 2). Further trials to separate the mixtures using various separation techniques failed; hence we are reporting the reduced compounds as is.

The ^1^H and ^13^C NMR spectral data analysis of the reaction products of rebaudioside B (**1**), rebaudioside C (**2**), and rebaudioside D (**3**) indicated the absence of the exocyclic double bond between *C*-16/*C*-17 position and the presence of their corresponding 17α and 17β methyl group isomers. The high resolution mass spectral (HRMS) data of the reduced products of rebaudioside B (**1**), rebaudioside C (**2**), and rebaudioside D (**3**) showed 2 amu more than their corresponding starting compounds which supported the hydrogenation.

### 2.2. Sensory Studies of Reduced Compounds **4**–**9**

The sensory evaluations of the synthetically reduced steviol glycosides **4**–**9** at a concentration of 500 ppm were performed against several control samples of 0.75%, 2%, 4%, 6%, and 7.0% sucrose equivalence (SE) in carbon treated (CT) water at room temperature (rt) as reported earlier [14]. Also, the sensory comparison of the mixtures **4**/**5**, **6**/**7**, and **8**/**9** against their original steviol glycosides was studied at 500 ppm using the controlled, multi-sip and swallow taste methods as described below. Sweetness evaluation results indicated that the sweet taste of the hydrogenated rebaudioside B derivatives (**4**/**5**) was reduced by over 75%, and that of the rebaudioside D derivatives (**8**/**9**) was reduced by about 25%, whereas the rebaudioside C derivatives (**6**/**7**) completely lost their sweetness after catalytic reduction (Table 1). These results indicated that the *C*16–*C*17 methylene double bond in steviol glycosides could be regarded as a pharmacophore essential for the sweetness property of these molecules [15,16]. Similar data has been identified earlier during our study on the catalytic hydrogenation of rubusoside, stevioside, and rebaudioside A [14].

### 2.3. Spectroscopy and Structural Characterization of Reduced Compounds **4**–**9**

Structural characterization of the reduced compounds **4**–**9** obtained by the catalytic hydrogenation of the three *ent*-kaurane diterpene glycosides isolated from *S. rebaudiana*; rebaudioside B (**1**), rebaudioside C (**2**), and rebaudioside D (**3**) was performed on the basis of one-dimensional (^1^H, ^13^C), two-dimensional [^1^H-^1^H correlation spectroscopy (COSY), ^1^H-^13^C heteronuclear multiple-quantum correlation (HMQC), ^1^H-^13^C heteronuclear multiple bond correlation spectroscopy (HMBC)] NMR and mass spectral data. The stereochemistry at the *C*-16 position was identified by comparison with their corresponding aglycone derivative NMR values reported in the literature [14,17–19], as well as enzymatic hydrolysis studies. The ^1^H-NMR and ^13^C-NMR values for all the protons and carbons in dihydrorebaudioside-B1/dihydrorebaudioside-B2 (**4**/**5**), dihydrorebaudioside-C1/dihydrorebaudioside-C2 (**6**/**7**), and dihydrorebaudioside-D1/dihydrorebaudioside-D2 (**8**/**9**) were assigned on the basis of COSY, HMQC and HMBC correlations. Further it was found that the ratio of 17α/17β reduced compounds was observed to be 3:2 for rebaudioside B (**1**); and 2:1 for rebaudioside C (**2**), and rebaudioside D (**3**). The ^1^H-NMR spectral data for the sp3 methyl groups and anomeric protons for the mixture of reduced compounds **4**–**9** are given in Table 2, whereas the complete assignments of their carbon values are given in Table 3.

## 3. Experimental Section

### 3.1. General

Melting points were measured using a SRS Optimelt MPA 100 instrument and are uncorrected. Infrared (IR) spectral data was acquired using a Perkin Elmer 400 Fourier Transform Infrared (FT-IR) Spectrometer (Atlanta, USA) equipped with a Universal Attenuated Total Reflectance (UATR, Atlanta, USA) polarization accessory, whereas NMR spectra were acquired on Varian Unity Plus 600 MHz instrument (Atlanta, USA) in C_5_D_5_N using standard pulse sequences. Chemical shifts were given in δ (ppm), and coupling constants were reported in Hz. HRMS and MS/MS data were generated with a Waters Premier Quadrupole Time-of-Flight (Q-TOF, New Jersey, USA) mass spectrometer equipped with an electrospray ionization source operated in the positive-ion mode and ThermoFisher Discovery OrbiTrap (New Jersey, USA) in the positive mode of electrospray. All the samples were diluted with water: acetonitrile (1:1) containing 0.1% formic acid and introduced via infusion using the onboard syringe pump. CT water was prepared by passing water through granular or block carbon material to reduce toxic compounds as well as harmless taste- and odor-producing chemicals.

### 3.2. Isolation of Reduced Steviol Glycosides **4**–**9**

#### 3.2.1. General Procedure for the Catalytic Hydrogenation of Steviol Glycosides **1**–**3**

Pd(OH)_2_ (50 mg) was added to a solution of each steviol glycoside **1***–***3** (2 g) in EtOH/H_2_O (8:2, 100 mL). The mixture was hydrogenated at ambient temperature for 5 days under hydrogen (H_2_) pressure at 55 psi. After each day, an aliquot of the reaction mixture was filtered through Celite and analyzed by high performance liquid chromatography (HPLC) for the absence of starting materials. At the end of hydrogenation (5 days), the reaction mixture was filtered through celite and concentrated under vacuum to afford a clear white product. The product was triturated with acetone, filtered and dried under vacuum at 50 °C for 48 h–72 h. The combined purity of each isomeric mixture **4**/**5**, **6**/**7**, and **8**/**9** was checked by HPLC and was found to be >97%.

Dihydrorebaudioside B1/Dihydrorebaudioside B2 (**4/5**). White powder; IR ν_max_: 3347 cm^−1^, 2928 cm^−1^, 2882 cm^−1^, 1726 cm^−1^, 1034 cm^−1^, 891 cm^−1; 1^H-NMR and ^13^C-NMR spectroscopic data see Tables 2,3, respectively; HRMS (*M* + NH_4_)^+^*m*/*z* 824.4282 (calcd. for C_38_H_66_NO_18_: 824.4280), (*M* + Na)^+^*m*/*z* 829.3838 (calcd. for C_38_H_62_O_18_Na: 829.3834).

Dihydrorebaudioside C1/Dihydrorebaudioside C2 (**6**/**7**). White powder; IR ν_max_: 3355 cm^−1^, 2932 cm^−1^, 2881 cm^−1^, 1722 cm^−1^, 1034 cm^−1^, 890 cm^−1; 1^H-NMR and ^13^C-NMR spectroscopic data see Tables 2,3, respectively; HRMS (*M* + NH_4_)^+^*m*/*z* 970.4864 (calcd. for C_44_H_76_NO_22_: 970.4859), (*M* + Na)^+^*m*/*z* 975.4418 (calcd. for C_44_H^72^NaO_22_: 975.4413).

Dihydrorebaudioside D1/Dihydrorebaudioside D2 (**8**/**9**). White powder; IR ν_max_: 3345 cm^−1^, 2920 cm^−1^, 2882 cm^−1^, 1724 cm^−1^, 1035 cm^−1^, 880 cm^−1; 1^H-NMR and ^13^C-NMR spectroscopic data see Tables 2,3, respectively; HRMS (*M* + H)^+^*m*/*z* 1131.5074 (calcd. for C_50_H_83_O_28_: 1131.5071), (*M* + NH_4_)^+^*m*/*z* 1148.5342 (calcd. for C_50_H_86_NO_28_: 1148.5336).

#### 3.2.2. General Procedure for the Enzymatic Hydrolysis of Reduced Steviol Glycoside Mixtures

**Figure 2:**
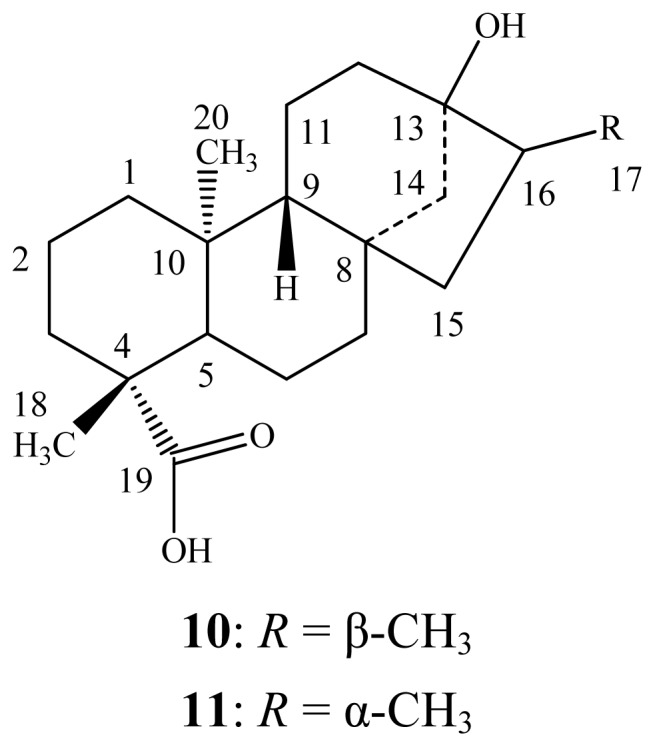
Structures of dihydrosteviol A (**10**) and dihydrosteviol B (**11**).

The mixture of each reduced steviol glycoside (250 mg) was dissolved in 0.1 M sodium acetate buffer, pH 4.5 (50 mL) and crude pectinase from *Aspergillus niger* (15 mL, Sigma-Aldrich, P2736) was added. The reaction mixture was stirred at 50 °C for 96 h. The product precipitated out during the reaction for all three mixtures **4**/**5**, **6**/**7** and **8**/**9** was identified as the same based on spectral data and TLC. The filtered compound was purified over silica gel column chromatography; elution with *n*-hexane/acetone (9.5:0.5) yielded a pure Compound **10** (18 mg, mp: 186 °C –190 °C) whereas elution with *n*-hexane/acetone (9.0:1.0) yielded another pure Compound **11** (13 mg, mp: 212 °C –214 °C). The two Compounds **10** and **11** (Figure 2) were identified as dihydrosteviol A and dihydrosteviol B by comparison of their physical and ^1^H-NMR spectral data with the literature values [17–19].

### 3.3. Sweetness Evaluation of the Reduced Steviol Glycoside Mixtures **4**–**9**

Sweetness evaluation of the reduced steviol glycoside mixtures was performed using sucrose as a control along with their original compounds. Extra fine cane sucrose from Domino (lot#11:09 6843 1A10) was used for preparation of control samples. Carbon-treated (CT) water was used for all preparations of control, standard steviol glycosides and their reduced compounds. The reduced steviol glycoside mixtures were prepared at 500 ppm for sensory evaluation by adding the non-moisture compensated mass into a 100 mL sample of CT water. The mixtures were moderately stirred at room temperature (rt), and the reduced steviol glycoside samples were then evaluated against several control sucrose samples at 0.75%, 2%, 4%, 6% and 7.0% SE in water at RT by experienced Research and Technology panelists at The Coca-Cola Company, Atlanta, USA, for any tasting quality determinations using the controlled, multi-sip and swallow taste methods shown below.

### 3.4. Multi-Sip and Swallow Taste Method

1. Take first sip (~1.8 mL) of a full medicine cup and swallow the control, wait for 15 s–25 s, then take the second sip and lock it into memory and wait for 15 s–25 s.
2. Taste the first sip of the experimental sample; wait for 15 s–25 s, then use the second sip to compare to the second sip of the control.
3. Repeat steps #1 and #2 for the third and fourth sips of the same control and experimental samples to confirm the initial finding.

## 4. Conclusions

In conclusion, six *ent*-kaurane diterpene glycosides **4**–**9** were synthesized by catalytic hydrogenation of the three natural products rebaudioside B (**1**), rebaudioside C (**2**), and rebaudioside D (**3**) using Pd(OH)_2_. The structures of all synthesized compounds were characterized on the basis of NMR (1D and 2D) and mass spectral data, enzymatic hydrolysis as well as in comparison with the data reported in the literature. This is the first report of the complete spectral characterization of the reduced compounds of rebaudioside B, rebaudioside C, and rebaudioside D.

## Figures and Tables

**Table 1:** Sensory evaluation of rebaudioside B (**1**), rebaudioside C (**2**) and rebaudioside D (**3**) *versus* catalytically hydrogenated steviol glycosides (**4**–**9**) at 500 ppm in CT water at RT.

Steviol Glycoside Type	Sensory Evaluation of Original Compound	Sensory Evaluation of Reduced Compound
Rebaudioside B (**1**)	Slow onset of sweetness, sweet lingering aftertaste, about 5%–6% sucrose equivalence	Weak sweetness, about 1% sucrose equivalence
Rebaudioside C (**2**)	Slow onset of sweetness, less sweet overall than sucrose, about 2%–3% sucrose equivalence	No sweetness, moderate astringency
Rebaudioside D (**3**)	Slow onset of sweetness, very clean, sweeter overall than sucrose, less sweet lingering aftertaste compared to sucrose, about 6%–7% sucrose equivalence	Slow onset of sweetness, no sweet lingering taste, about 5%–5.5% sucrose equivalence

**Table 2:** ^1^H-nuclear magnetic resonance (NMR) chemical shift values for reduced compounds **4**–**9** recorded in C_5_D_5_N a**–**c.

Position	4	5	6	7	8	9
17	1.17 (d, 6.6, 1*H*)	1.34 (d, 6.3, 1*H*)	1.19 (d, 6.6, 1*H*)	1.39 (d, 6.4, 1*H*)	1.13 (d, 6.5, 1*H*)	1.17 (d, 6.4, 1*H*)
18	1.16 (s, 3*H*)	1.18 (s, 3*H*)	1.24 (s, 3*H*)	1.27 (s, 3*H*)	1.14 (s, 3*H*)	1.15 (s, 3*H*)
20	1.19 (s, 3*H*)	1.32 (s, 3*H*)	1.29 (s, 3*H*)	1.28 (s, 3*H*)	1.41 (s, 3*H*)	1.42 (s, 3*H*)
Sugar I-1′	5.04 (d, 6.6, 1*H*)	5.03 (d, 6.4, 1*H*)	6.15 (d, 6.8, 1*H*)	6.16 (d, 6.5, 1*H*)	6.88 (d, 6.4, 1*H*)	6.84 (d, 6.5, 1*H*)
Sugar II-1″	5.33 (d, 6.4, 1*H*)	5.36 (d, 6.3, 1*H*)	5.09 (d, 6.7, 1*H*)	5.06 (d, 6.4, 1*H*)	5.51 (d, 6.6, 1*H*)	5.54 (d, 6.4, 1*H*)
Sugar III-1‴	5.47 (d, 6.1, 1*H*)	5.52 (d, 6.4, 1*H*)	5.95 (d, 6.5, 1*H*)	5.77 (d, 6.8, 1*H*)	5.50 (d, 6.6, 1*H*)	5.58 (d, 6.5, 1*H*)
Sugar IV-1″″			6.53 (d, 1.8, 1*H*)	6.86 (d, 1.6, 1*H*)	5.38 (d, 6.4, 1*H*)	5.42 (d, 6.6, 1*H*)
Sugar V-1‴″					6.33 (d, 6.4, 1*H*)	6.31 (d, 6.2, 1*H*)
Sugar III-6‴			1.65 (d, 6.1, 3*H*)	1.74 (d, 6.4, 3*H*)		

a Assignments made on the basis of correlation spectroscopy **(**COSY), heteronuclear multiple-quantum correlation (HMQC) and heteronuclear multiple bond correlation spectroscopy (HMBC) correlations;

b Chemical shift values are in δ (ppm);

c Coupling constants are in Hz.

**Table 3:** ^13^C-NMR chemical shift values for reduced compounds **4**–**9** recorded in C_5_D_5_N a, b.

Position	4	5	6	7	8	9
1	40.2	40.2	41.2	41.2	41.1	41.1
2	20.2	20.4	20.2	20.1	20.6	20.4
3	38.9	38.8	38.9	39.0	38.6	38.3
4	44.3	43.2	44.4	43.4	44.6	42.9
5	57.5	56.0	58.1	57.9	58.0	57.9
6	23.3	23.6	22.7	23.0	23.1	22.9
7	41.5	40.3	41.7	40.1	41.2	40.1
8	44.1	43.1	44.3	43.0	43.7	42.4
9	56.0	50.8	56.6	54.7	55.6	54.9
10	40.2	40.3	40.3	40.3	40.1	40.3
11	20.5	20.7	20.3	20.6	20.6	20.7
12	35.4	44.0	35.3	44.2	35.9	44.0
13	88.6	88.3	86.2	86.2	88.2	88.1
14	47.8	50.8	47.3	50.2	47.7	50.9
15	47.8	44.3	47.2	44.9	47.6	44.8
16	41.5	38.7	41.2	39.0	41.1	38.9
17	16.3	16.5	14.2	19.7	14.4	17.2
18	29.7	29.8	28.6	28.6	29.4	29.8
19	180.6	180.5	177.8	177.7	176.5	176.4
20	15.9	16.1	15.8	16.0	15.7	15.9
1′	98.6	98.8	96.2	95.7	96.2	96.2
2′	78.8	78.7	75.5	75.4	81.4	81.0
3′	85.7	86.6	79.0	79.2	78.8	78.7
4′	72.1	72.2	71.4	71.4	71.5	71.4
5′	77.0	77.0	78.5	78.6	78.5	78.6
6′	62.9	62.8	62.5	62.5	63.1	63.3
1″	105.4	105.0	98.3	96.8	94.2	94.3
2″	74.6	74.6	78.4	78.6	79.1	79.2
3″	77.8	77.8	87.1	86.1	86.0	86.7
4″	72.1	72.3	70.6	70.5	71.2	71.1
5″	79.0	79.2	75.6	75.4	77.2	77.0
6″	62.9	62.8	62.7	62.8	62.8	62.9
1‴	105.5	105.3	103.1	102.1	105.1	104.8
2‴	75.6	75.7	71.7	71.6	75.7	75.9
3‴	81.9	81.3	72.9	72.8	78.7	78.6
4‴	72.1	72.1	73.1	73.2	72.3	72.1
5‴	79.1	79.0	70.3	70.0	79.0	79.1
6‴	63.1	63.3	19.4	19.3	62.5	62.8
1″″			105.0	104.9	105.3	105.9
2″″			74.9	74.7	74.3	74.4
3″″			79.8	79.7	79.8	79.9
4″″			72.2	72.1	72.2	72.1
5″″			79.0	78.9	79.6	79.7
6″″			63.6	63.1	63.2	63.1
1‴″					106.1	105.4
2‴″					76.8	76.7
3‴″					78.9	78.8
4‴″					72.3	72.1
5‴″					81.8	81.4
6‴″					63.5	63.5

a Assignments made on the basis of COSY, HMQC and HMBC correlations;

b Chemical shift values are in δ (ppm).
